# Synergistic effect of electrolyzed oxidized water (EO) and peroxyacetic acid on plasmid-mediated quinolone resistance genes of *Pseudomonas aeruginosa*

**DOI:** 10.1007/s11274-025-04384-w

**Published:** 2025-06-14

**Authors:** Hanan R. Ghanayem, Khalid Tolba, Mariam M. G. El-Shemy, Fawzia A. El-Shenawy

**Affiliations:** 1 Food Hygiene Department, Animal Health Research Institute (AHRI), Agriculture Research Center (ARC), Tanta Lab, Tanta City, Egypt; 2 References Lab. for Food Safety, Animal Health Research Institute (AHRI), Agriculture Research Center (ARC), Dokki-Giza City, Egypt; 3 Bacteriology Unit, Animal Health Research Institute (AHRI), Agriculture Research Center (ARC), Tanta Lab, Tanta City, Egypt

**Keywords:** *P. aeruginosa*, Plasmid-mediated quinolone resistance (PMQR) genes. Electrolyte oxidized water (EOW), Per-acetic acid

## Abstract

*Pseudomonas aeruginosa* displays major biofilm formation, food spoilage, and significant losses for the food industry. It extremely exhibits inherent resistance to various antibiotics, including quinolones, which can spread via plasmids. The present study investigates the prevalence of biofilm-forming *P. aeruginosa* in meat products and assesses its antibiotic resistance patterns. Evaluating the effectiveness of electrolyzed-oxidized water (EOW) and per-acetic acid (PAA) alone or in a combination against *P. aeruginosa* biofilm formed on stainless steel (SS) surfaces. Besides, the effect on quinolone-resistance (*qnr*) gene expression level using real-time PCR. *P. aeruginosa* was isolated from 24.6% of the total tested samples (37/150), with a significant variation observed regarding the contamination levels of the tested products. 36.3% of the isolates demonstrated robust biofilm production, with 82.3% displaying multi-drug resistance (MDR). Isolates revealed complete susceptibility to amikacin (100%) and gentamycin (82%). Quinolone resistance was observed in 23% and 17% of the isolates for ciprofloxacin and levofloxacin, respectively. 80% (4/5) among the confirmed isolates were enclosed the plasmid *qnr* genes. The genes *psl*A and *gyr*A were detected in 40% and 60%, respectively. EOW, particularly when combined with PAA, reveals strong antibacterial activity against *P. aeruginosa* and greatly decreases its count. Moreover, it can eliminate its biofilm within 20 min of exposure and significantly decrease the expression levels of quinolone-resistant genes. In conclusion, PAA and EOW are effective agents for biofilm control on SS surfaces, particularly when used in combined form, and could be more effective in combating resistant bacterial infections and controlling the spread of PMQR.

**Supplementary Information:**

The online version contains supplementary material available at 10.1007/s11274-025-04384-w.

## Introduction

*Pseudomonas aeruginosa* are dangerous bacteria that can be found in a variety of surroundings, such as soil, water, air, animal tissues, and plants. (Cross et al. 1983). Meat products can be damaged by *P. aeruginosa* due to its proteolytic, lipolytic, and saccharolytic activity. (Alimi et al. 2022). Certainly, microbial decomposition is the public and mutual cause of food spoiling, and it costs the food industry a significant financial losses (Dogan and Boor 2003). Meat and its products for example sausage, beef-burgers, as well as luncheons, are the best and widely spread because they are high in nutrients, tasty, easy, and quick to prepare. However, meat and its products are extremely vulnerable to microbial deterioration, which can increase the risk of infection and even death, particularly in developing countries. (Wickramasinghe and Ravensdale 2019). Stainless steel (SS) surfaces are broadly used in the equipment of the food processing. Due to its ability to withstand corrosion, it is regarded as an exceptional material for food processing equipment. (Tantratian et al. 2022).

The construction of biofilm is known as the very predominant mechanism for the adaptive resistance of *Pseudomonas aeruginosa*, resulting in determined infection (Taylor et al. 2014). A biofilm consists of a group of bacteria that are arranged on a living or biological surface and stick together. (Das et al. 2013). Biofilms begin to produce immediately if the bacterial cells catch themselves conclusively on a surface with each other and are surrounded with a self-produced and/or developed exopolymeric matrix (Barraud et al. 2015). In biofilms, this formed polymeric matrix restrains microorganisms and supports enzymatic reactions, quorum sensing, and horizontal gene transfer (Sutherland 2001). Moreover, the formed biofilms provide powered stability against sanitizers, antimicrobial agents, and host immune response (Stewart and Costerton 2001; Kumar et al. 2013). The *Psl*A gene promotes the growth of biofilms and helps bacteria to withstand antibiotics and the host immune system (Emami et al. 2015). Multidrug-resistant phenotypes can be developed in *P. aeruginosa* through the attainment of multiple current resistance mechanisms on mobile genomic elements (Lister et al. 2009). In comparison to other bacteria, *P. aeruginosa* is difficult to eradicate as it exhibits increased inherent resistance to an extensive variety of antibacterials, as well as quinolones (Breidenstein et al. 2011). *P. aeruginosa* may involve acquired resistance using horizontal transfer of genetic elements comprising plasmids (Breidenstein et al. 2011). Plasmids enclose numerous resistance cassettes, so this can increase antibiotic resistance (Raposo et al. 2016). Quinolone resistance mediated by plasmid is a significant public mechanism for resistance amongst Gram-negative bacteria (Taha et al. 2019). The *qnr* genes (which code for *qnr* proteins) and include *qnr*A, *qnr*B, and *qnr*S have been identified, and they counteract the blockage effects of quinolone antibiotics on the bacterial enzymes, such as DNA gyrase (Rodríguez-Martínez et al. 2016). Therefore, it is essential to search for and develop sanitizers that can effectively combat biofilms. The spread of chemical antibacterial agents is a quiet approach for controlling or decreasing food-borne pathogens during food processing. Peroxyacetic acid (PAA) is a carbon-based liquefied peroxide, colorless with a little pH, a sharp-tasting, vinegar-like product that can develop antibacterial activity against bacteria. PAA is permitted by the US Food and Drug Administration (FDA) and the United States Department of Agriculture-Food Safety and Inspection Services (USDA-FSIS) to be considered as a generally probable and safe antimicrobial agent for meat and chicken, in addition to egg products (Li et al. 2020; Stearns et al. 2022). Additionally, electrolyzed water (EW) is a green technology that uses electrolysis to physically separate the anode and cathode while permitting certain ions to pass through a semi-permeable membrane in a diluted sodium chloride solution. In many nations, EW is primarily utilized in food manufacturing as an antibacterial, disinfectant, and sanitizer (Hricova et al. 2008; Takeda et al. 2023). Therefore, this study was designed to (i) determine the incidence rate of *P. aeruginosa* in meat products, assess the biofilm-forming ability of *P. aeruginosa*, and determine the antibiotic resistance profile of the isolates. (ii) Detection of the common biofilm-associated gene pslA and PMQR genes; (iii) Investigate the inhibitory activity of electrolyzed-oxidized water (EOW) and peroxyacetic acid (PAA) on the biofilm and the expression level of the *psl*A gene and quinolone-resistant genes.

## Materials and methods

### Samples collection

Between March and June 2024, a total of 150 meat product samples, including minced meat, beef burgers, and luncheons (50 from each), were collected from Gharbia Governorate, Egypt. The total samples were obtained separately and directly transported to the laboratory in an icebox for bacteriological examination.

### Isolation and identification of *P. aerogonosa*

Microscopic examination of Gram staining and biochemical identification tests, including the catalase test. Twenty-five grams from every sample were homogenized within peptone water (225 ml); samples were later incubated at 37 °C for 24 h then a loop full from the samples was spread over the surface of blood agar, nutrient agar, and *Pseudomonas* Cetrimide-agar (OxoidTM) according to (Quinn et al. 2011). After incubation of the cultured plates, the suspected-separate pure colonies were identified through TSI, oxidase test, production of Pyoverdin, and growth at 42 °C and 5 °C, which were dependent to confirm the identification of *p. aeruginosa* according to (Roberts and Greenwood 2008; Alikhani et al. 2014).

### Phenotypic detection of biofilm forming ability

#### Three distinct methods were used to asses the capacity of isolated strains for biofilm production

The tube method is simple and sensitive for the detection of biofilm formation (Reddy 2017). Polystyrene test tubes with round bottoms were used for the test; isolated *P. aeruginosa* strains were cultured on trypticase-soy broth (TSB) (OXOID) containing 1% glucose, incubated at 37^0^C for for 24 h, then the planktonic cell culture was discharged from the tubes and washed two times using phosphate-buffered saline (PBS) followed by staining with crystal violet (0.1%) for 20–30 min. Then, stained tubes were rinsed twice with phosphate buffer saline. After that, the tubes were left to air dry (Sultan and Nabiel 2018). The experiment was achieved for triplicates. Positive tubes for biofilm production had stained adhesive layers lining the wall and bottom. Meanwhile, tubes with stained rings at the interface of liquid air were considered non-biofilm producers. Biofilm producers were characterized as weak, moderate, and strong biofilm producers according to the intensity of biofilm observed (Deka 2014; Sagar et al. 2023).

#### Congo red agar assay

On CRA media made of 37-gram brain heart infusion (BHI) agar (OXOID, UK) 10 g agar and 5-gram sucrose, all dissolved in one liter of distilled water and autoclaved, 0.8 g of sterilized Congo red stain (OXOID, UK) was prepared, and when cooled, the sterile BHI agar was added. Pure colonies were spread over Congo red agar plates and incubated at 37 °C overnight Black colonies with crystalline consistency are considered biofilm-producers while the red colonies are non-biofilm producers (Sultan and Nabiel 2018). Each isolate was cultured for triplicates.

### Micro-titre tissue culture plate (TCP) method

Fresh culture from the obtained isolates was conveyed onto fresh broth media with a dilution of 1/100. A flat-bottom polystyrene tissue culture plate (TCP) was used. A polystyrene tissue culture plate (TCP) with a flat bottom was employed. 200 µL of each strain’s diluted culture was put in to each well. As a negative control, one well was filled with sterile broth. Following a 24-hour incubation period at 37 °C, the fillings in each well were carefully tapped out and rinsed away three times with a 0.2 mL PBS solution to get rid of any free-floating bacteria. Sodium acetate (2%) was used to fix the biofilm, and crystal violet (0.1%) was used to stain it. The plate was left to dry after using deionized water to remove the excess stain (Sultan and Nabiel 2018). The absorbance was measured at 630 nm using a microplate ELISA reader (MR-96 CLINDIAG Device) at the Micro Analysis Unit, Faculty of Science, Tanta University after adjustment to zero with negative control as blank. The experiment was performed in triplicates. The average OD values were considered for all tested strains and negative controls. The optical density cut-off value (ODc) was established, ODc = average OD of the negative controls + (3 × SD of negative control). If average OD ≤ ODc, considered as a non-biofilm producer; if ODc < OD ≤ 2 ODc, weak biofilm producer; 2 ODc < OD ≤ 4 ODc, indicates moderate biofilm was produced; if 4 ODc < OD, considered as a strong biofilm producer (Stepanović et al. 2007; Kabir et al. 2024).

### Antimicrobial susceptibility testing

Antimicrobial susceptibility test was performed by the disc diffusion method according to the National Committee for Clinical Laboratory Standards (NCCLS) also, the inhibition zones were measured and interpreted according to Clinical and Laboratory Standards Institute criteria (CLSI 2020). Briefly, a standardized inoculum was cultured above the surface of Mueller–Hinton (MH) agar (Merck, Germany), then antibiotic disks were distributed on the surface of the agar. Diameters of the inhibition zone around the disks was measured after incubation at 37 °C ffor 24 h. The following antibiotic disks (Yenimahalle-Ankara/Turkey) were used: β-lactam combination (Amoxicillin/Clavulanate AMC; 30 µg), Cephems (Ceftazidime CAZ;30 µg, and Cefotaxim CTX;30 µg), carbapenems (Meropenem MEM;10 µg), Fluoroquinolones (Ciprofloxacin CIP;5 µg, and Levofloxacin LEV; 5 µg), Aminoglycosides (Gentamicin GN; 10 µg, Amikacin AK;30 µg), and Sulfamethoxazole /Trimethoprim SXT;25 µg. The multidrug-resistant (MDR), extensively drug-resistant (XDR) isolates characterized according to the criteria defined by (Magiorakos et al. 2012). Each antimicrobial susceptibility testing was simultaneously done in triplicates.

### PCR assay for confirmation of *P*. *aeruginosa*, detection of plasmid mediated quinolone resistant (PMQR) genes, *gyr*A and biofilm gene(*psl*A)

Five *P. aeruginosa* isolates were selected based on strong biofilm production and phenotypic pattern for quinolone resistance and were suspected to conventional uniplex PCR assay for molecular identification based on specific primer sequence of 16 S rRNA. Then the confirmed isolates were screened for the existence of PMQR genes (*qnr*A, *qnr*B^a^ and *qnr*S) besides, the *gyr*A and biofilm-associated gene (*psl*A). DNA extraction from isolates was achieved using the DNA Mini kit for QIAamp (Qiagen, Germany, GmbH, with Catalogue no. 51304) with little modification from the manufacturer’s recommendations. Plasmid DNAs from selected strains were extracted using the Thermo-Scientific Gene JET for the Plasmid Miniprep Kit (Thermo, Germany) for quinolone resistance genes. PCR amplification was done with a PTC-100 programmable thermal cycler (Peltier-Effect cycling, MJ, UK) when adjusting the absolute volume of the reaction mix. The primers (Metabion, Germany) used for amplifications and cycling conditions are listed in Tables 1 and 2. The PCR-amplified products were separated by electrophoresis on agarose gel (1.5%).

**Table 1:** Oligonucleotide primer sequences

Gene	Sequence	Amplified product	Reference
16 S rRNA *P. aeruginosa*		956 bp	(Spilker et al. 2004)
	GGGGGATCTTCGGACCTCA		
*psl*A	TCCTTAGAGTGCCCACCCG	656 bp	(Ghadaksaz et al. 2015)
	TCCCTACCTCAGCAGCAAGC		
*gyr*A	TGTTGTAGCCGTAGCGTTTCTG	358 bp	(Singhal et al. 2016)
	CAGCTACATCGACTATGCGA		
*qnr*A	ATTTCCCTCA GCATCTCCA	580 bp	(Cattoir et al. 2007)
	AGA GGA TTT CTC ACG CCA GG		
*qnr*Ba	TGC CAG GCA CAG ATC TTG AC	264 bp	(Cattoir et al. 2007)
	GGM ATH GAA ATT CGC CAC TG		
*qnr*S	TTT GCY GYY CGC CAG TCG AA	428 bp	(Cattoir et al. 2007)
	GCA AGT TCA TTG AAC AGG GT		

**Table 2:** Cycling conditions used for amplifications

Target gene	Primary denaturation	Amplification				Final extension
		Secondary denaturation	Annealing	Extension	No. of cycles	
16s RNA	94˚C, 5 min.	94˚C, 1 min	58˚C, 1 min	72˚C 1 min	30	72˚C 5 min.
*pslA*	95˚C, 3 min.	95˚C, 1 min	60˚C, 40 s	68˚C 1 min	35	68˚C 5 min.
*gyrA*	94˚C, 5 min.	94˚C, 30 s	60˚C, 30 s	72˚C 30 s	35	72˚C 7 min.
*qnrA*	94˚C, 5 min.	94˚C, 30 s	54˚C, 45 s	72˚C 45 s	35	72˚C 5 min
*qnrB* ^a^	94˚C, 30 s.	94˚C, 40 s	54˚C, 40 s	72˚C 40 s	40	72˚C 10 min.
*qnrS*	94˚C, 30 s.	94˚C, 40 s	54˚C, 40 s	72˚C 40 s	40	72˚C 10 min.

### Antibacterial activity of EOW and PAA against *P. aeruginosa*

Antibacterial susceptibility test was done by way of the agar-well diffusion method using Mueller–Hinton (MH) agar plates according to (Hossain 2023). A pure refresh culture strain of *P. aeruginosa* (resistant against levofloxacin and ciprofloxacin) was adjusted to the standard and then spread over the plate of MH agar. After that, wells were pierced inside the agar using a sterilized 6 mm diameter puncher. EOW(pH, 2.1) alone, PAA with a concentration of 0.1% and 0.3% as well as a combined mix of EOW + 0.1% PAA, were freshly prepared with sterile distilled water. Fifty µl of each concentration was separately distributed with a sterile micropipette into the different wells. Tested inoculated plates were upright incubated at 37° C for 16–18 h, the inhibition zone diameters were recorded from the edge in millimeters (mm), and the results were recorded as; 0–5 mm was considered as no inhibition (resistant); 6–9 mm was a moderate inhibition; while 10–14 mm was stated as a strong inhibition, and more than 15 mm was distinguished as an actual strong inhibition (sensitive (Ali et al. 2021). The test was performed in triplicates, and the mean of the obtained values was recorded.

### Experimental part to detect disinfectant activity of EOW and PAA against the tested strains of *P. aeruginosa* and their biofilm growth on stainless steel (SS) surface

#### Preparation of bacterial strain

PCR confirmed *P. aeruginosa* strains recovered from meat product samples (minced meat, luncheon, and beef burger), had the biofilm-forming ability, and contained PMQR genes. A pure refreshed strain was standardized to a 0.5 McFarland concentration of 10^8 cfu ml − 1 (8 log 10 CFU/ml).

#### Preparation of stainless steel (SS) surfaces

The stainless steel surfaces, similar to that used in the food processing industry, were prepared and cleaned according to the method described by Rivas et al. (2007) with some modifications, they were firstly saturated with acetone for 30 min, then, washed away with distilled water and this process was repeated twice. After that, they were autoclaved at 121 °C for 15 min and then divided into six groups.

#### Preparation of EOW and PAA concentrations

One liter of drinking potable water was used with the addition of 0.2% of sodium chloride, passing a current of 9–10 V-amber (VA) through the prepared solution via an electrolysis cell containing two poles of anode (+) and cathode (-) for 10 min. Latter in the electrolysis process, the pH reaches (2.1). Furthermore, PAA was prepared at a concentration of 0.1% and 0.3%.

### Estimated no. (CFU/Cm^2^) of *P. aeruginosa* attached to SS surfaces groups and application of used treatments

The prepared *P. aeruginosa* strain was flooded onto the six prepared SS surfaces in an area diameter of (2 × 5 cm^2^), left for 30 min. for successful attachment, then the SS surfaces of the zero group (G0) was washed with DW (control–ve group) and used to estimate the initial contamination of *P. aeruginosa*, the 1st and 2nd SS groups were washed using 0.1% & 0.3% PAA, respectively, the 3rd SS group was treated using EOW (pH 2.1), while the SS of the 4th & 5th groups was washed with a mixture of EOW + PAA (0.1% and 0.3%) for the latter, respectively. Washing time continued for two minutes, followed by the removal of the excess treatment solutions from all groups. The adhered cells were then collected by rubbing the surfaces with sterile moistened swabs, and the number of *P. aeruginosa* was calculated.

### Biofilm formation

Bacterial culture was allowed to form a biofilm on SS discs according to Ripolles-Avila et al. (2018) with modification. Sterile SS discs were immersed in a petri dish that contained a solution of 20 ml nutrient broth containing 3 ml of the standard bacterial inoculum (10^8^ CFU/ml). The petri dishes were incubated at 37^0^ C for 48 h to permit the biofilm formation. The discs were then removed and washed with sterile saline PBS to remove the excess adhered cells. The biofilm was removed from the discs with sterile moistened swabs, which were then placed into 9 ml sterile peptone-water-containing tubes. Serial dilutions were then carried out on the solutions, and from each serial, 0.1 ml was plated on selective *pseudomonas* agar media. The plates were incubated at 37^o^C for 24 h. The biofilm population was quantified by enumerating viable colonies, and the results were expressed as log CFU/cm^2^ in the control-ve group (Ripolles-Avila et al. 2020).

### Effect of EOW and PAA on *P. aeruginosa* cells and its formed biofilm

#### Effect on *P. aeruginosa* count (log_10_ CFU/cm^2^)

After flooding the SS coupons in *P. aeruginosa* broth culture for a contact time of 30 min, it was rinsed twice with a sterile phosphate buffer and then exposed to treatment: Groups one and two were soaked in a petri dish containing 5 ml of PAA 0.1% and 0.3%, respectively; group three was soaked in a petri dish containing 5 ml of EOW (PH 2.1), while the 4th and 5th groups were soaked in 5 ml of a combined mix of EOW and PAA (0.1% & 0.3%), respectively. After 1, 2, 5, and 10 min of contact time, the adhered cells were collected by rubbing the surfaces with sterile, moistened swabs. The swabs were then suspended in 9 ml of sterile peptone water. The solutions were then subjected to serial dilutions. From each dilution, 0.1 ml was spread over plates of *P. aeruginosa* selective media agar, incubated at 37 °C for 24 h. The numbers of adhered cells were counted and expressed as log CFU/cm² (Ripolles-Avila et al. 2020).

#### Effect on *P. aeruginosa* biofilm

After forming the biofilm, the stainless steel coupons were rinsed twice with sterile phosphate buffer. They were then treated as follows: The 1st & 2nd groups were soaked in Petri dishes containing 5 ml of PAA (0.1% and 0.3%) respectively. Meanwhile, the 3rd Group was saturated with 5 ml of EOW (PH 2.1). However, the 4th & 5th groups were soaked in 5 ml of a pooled mixture of EOW + PAA (0.1% and 0.3%), respectively. They were soaked for 1, 2, 5, 10, 20 and 30 min. The adhered stacked cells were collected by rubbing the surfaces with sterile swabs that were then transferred to tubes containing 9 ml of sterile peptone water. The obtained solutions were subjected to serial dilutions, and 0.1 ml from all dilutions were streaked over *pseudomonas* agar media. The plates were incubated at 37 °C for 24 h, and the total counts of arranged cells were expressed as log CFU/cm^2^. The activity of EOW and PAA was determined by calculating the *P. aeruginosa* count log CFU/cm^2^ of treated groups (EOW & PAA) standardized with the log CFU/cm^2^ of the negative control (Maraques et al. 2007). All parts of the experiment were performed in triplicates.

### Consequence of treatments on the expression level of quinolone-resistant and biofilm-associated genes using real-time (RT–-PCR) assay

RT-PCR was considered to perceive the impact of treatment with EOW and PAA on the average expression levels of quinolone-resistant genes and biofilm-associated *psl*A gene. After treatment of samples: Treated 1 (group treated with PAA 0.1%), Treated 2 (group treated with EOW), and Treated 3 (group treated with a mix of EOW + PAA 0.1%) followed by incubation of treated samples at 37°C for 24 h. After that, RNA extraction from treated and control non-treated samples was applied using the QIAamp RNeasy Mini kit (Qiagen, Germany, GmbH). The sequence primers were provided by Metabion (Germany) and are recorded in Table 1. The primer sets were operated on a 25-µl reaction comprising 12.5 µl of the 2x HERA-SYBR^®^ Green-RT-qPCR Master Mix (Willowfort, UK); the reaction was completed in a StepOne real-time PCR machine in the Biotechnology Unit, Animal Health Research Institute, Zagazig Branch, Egypt. For assessment, the variation in gene expression levels on the RNA of the different groups, the average expression levels of the tested genes were standardized using 16SrRNA as housekeeping, and the CT of each group was paralleled with that of the positive-control group according to the “ΔΔCt” method. (Yuan et al. 2006).

### Statistical analysis of data

The data was analyzed and visualized using R software (R core Group, 2022; version 4.2.0). To assess the differences in the incidence rate of *P. aeruginosa* among different sources of isolation, Pearson’s chi-square test was employed. A mosaic plot with color case observed frequencies was created using the ggstatsplot and ggplot2 packages. Visualization of resistance rates of different antibiotics against *P. aeruginosa* isolates was done using the ggplot2 package, with violin and bar plots. Normality testing using the Shapiro-Wilk test was performed, followed by either one-way ANOVA (for parametric data) or the Kruskal-Wallis rank test (for nonparametric data), followed by Tukey’s HSD for multiple comparisons. Ranked Kruskal-Wallis test was directed to compare the mean of the multiple antibiotic resistance (MAR) index of the strains recovered from different meat products. Spearman’s correlation was estimated and visualized using the Hmisc and corrplot packages. The level of significance was recognized at *p*-value < 0.05.

## Results

### Incidence of *P. aeruginosa*

**Fig. 1:**
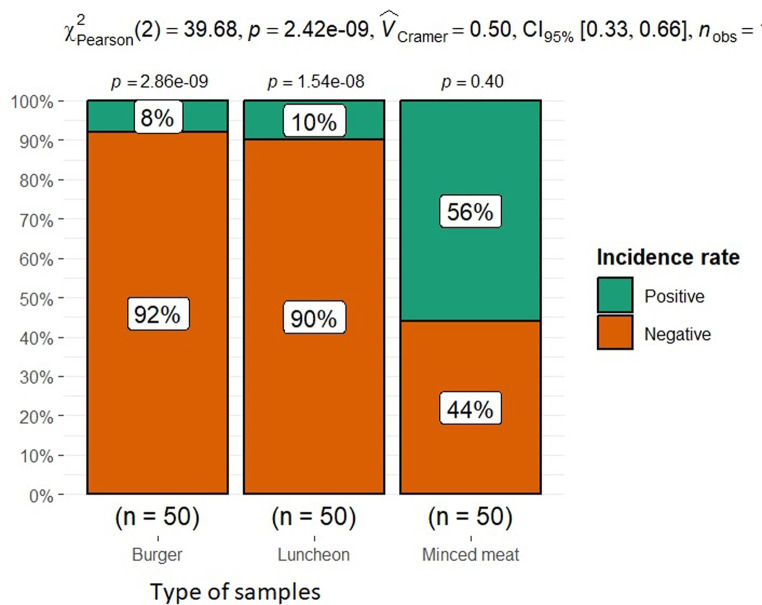
Mosaic plot with coloured cases for the observed frequencies in the incidence rate of *p. aeruginosa*

*P. aeruginosa* was isolated from the total tested samples with an incidence rate of 24.6% (37|150). The isolation rate from minced meat was 56% (28/50), from burgers was 8% (4/50), while the isolation rate from luncheon was 10% (5/50). The incidence rate of *P. aeruginosa* was significantly varied among the different meat product sources (𝑝-value ≤ 0.05) Fig. 1.

### Phenotypic detection of biofilm forming ability

The three distinct biofilm detection techniques were used to assess each strain isolate’s capacity to produce biofilms. While 51% of the isolates generated robust biofilm, and 29% of *P. aeruginosa* isolates were found to produce intermediate biofilm by the TM assay. However, 89.9% of the isolates were found to create biofilms using the TCP technique, with 36.3% producing strong biofilms, 40% producing intermediate biofilms, and 13.6% producing weak biofilms. 38% of the isolates produced biofilms, according to the development of CRA. Weak, intermediate, and strong biofilm production levels were more precisely categorized using the TCP approach. Individual images for phenotypic detection of biofilm-forming ability were presented in Figs. S1. 1- S1. 4. inaddition to Table S1.

### Antimicrobial susceptibility

**Fig. 2:**
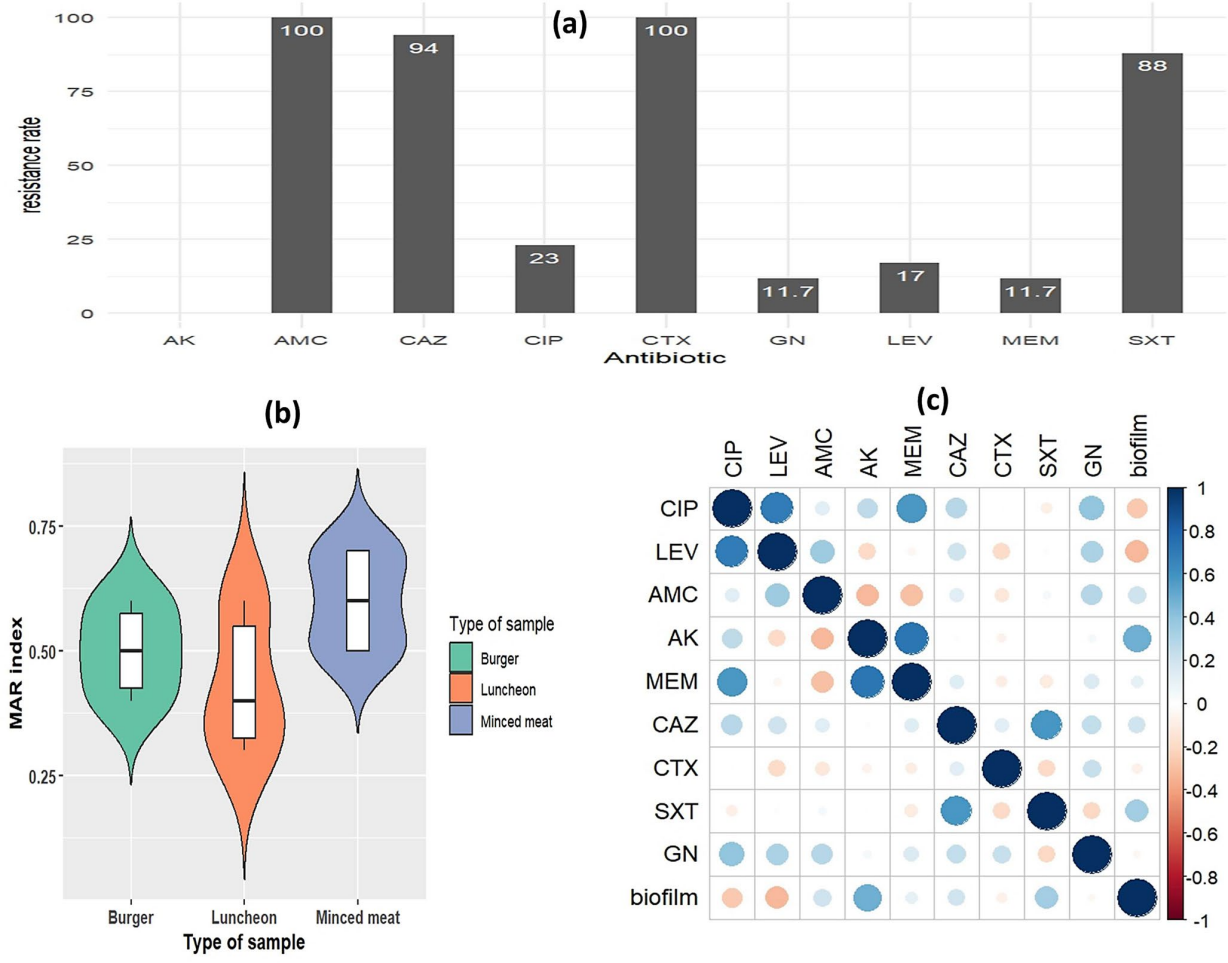
(**a**): Resistance rate of *p. aeruginosa* isolates to different antibiotics. (**b**): The violin plot showed variation in multiple antibiotic resistance in isolates. (**c**): Spearman’s correlation plot among different antibiotic-resistant phenotypes and biofilm production. Correlation coefficients are shown as colours on the scale (positive: blue, negative: red). AMC: amoxicillin/clavulanate, CAZ: ceftazidime, CTX: cefotaxim, MEM: meropenem, CIP ciprofloxacin, LEV: levofloxacin, GN: gentamicin, AK: Amikacin and SXT: sulfamethoxazole /trimethoprim

According to the interpretation results of antimicrobial susceptibility testing, the isolates revealed complete (100%) resistance to amoxicillin/clavulanate and cefotaxime, followed by ceftazidime (94%) and lower resistance to sulfamethoxazole/trimethoprim (88%). Only 17% of the isolates showed resistance to levofloxacin, while 23% showed resistance to ciprofloxacin. The isolates displayed complete susceptibility to amikacin (100%), while 82% of the isolates showed sensitivity for gentamycin and meropenem (Fig. 2). From the total isolates, 82.3% revealed MDR with 9 changed patterns for resistance with MAR index ranging from 0.33 to 0.77. Non-significant variation in the MAR index of isolates from the different sources, and the highest MAR index was detected in isolates from minced meat; however, the isolates improved from luncheons showed a lower MAR index (0.33) (Fig. 2). The majority of quinolones-resistance was perceived in minced meats and burger isolates. Spearman’s correlation coefficient investigation revealed a positive significant correlation between levofloxacin and ciprofloxacin-resistant phenotypes in the examined isolates (*p*-value = 0.0018, *R* = 0.70) and also, a positive correlation concerning amikacin and meropenem (p-value = 0.0010, *R* = 0.72), and a moderately significant correlation between meropenem and ciprofloxacin(p-value = 0.0160, *R* = 0.62), between sulfamethoxazole /trimethoprim and ceftazidime (p-value = 0.0128, *R* = 0.59). Meanwhile, non-significant or no correlation among other antibiotic-resistant phenotypes (Fig. 2). Statistical investigation indicated that MDR was unrelated to biofilm density (X-squared = 0.55, 𝑝-value = 0.45).

### Detection of plasmid-mediated quinolone-resistant (PMQR) genes and biofilm-associated gene *psl*A

**Fig. 3:**
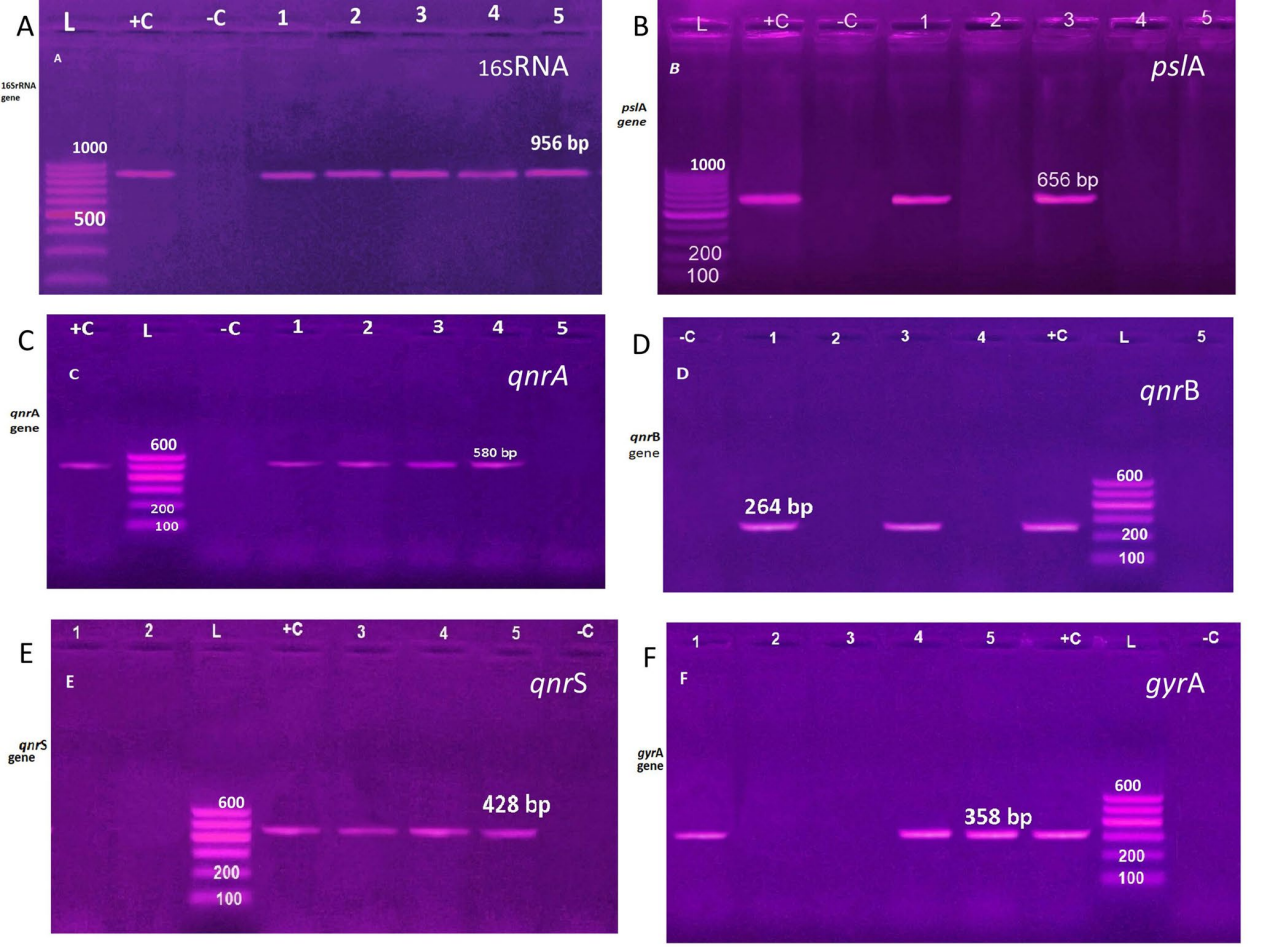
Agarose gel electrophoresis for uniplex conventional PCR amplification of (**A**): amplification of 16SrRNA gene for identification of *P. aeruginosa* isolates at 956 bp, (**B**): amplification of *psl*A gene at 656 bp, (**C**): amplification of *qnr*A gene at 580 bp, (**D**): amplification of *qnr*B gene at 264 bp, (**E**): amplification of *qnr*S at 428 bp and (**F**): amplification of *gyr*A gene at 358 bp. Lane L: ladder, Lane 1–5 for samples

Among the tested *P. aeruginosa* isolates, 80% (4/5) enclosed the plasmid *qnr*A gene, 60% (3/5) of the isolates enclosed the *qnr*S gene, while *qnr*B^a^ was perceived only in 40% (2/5) of the examined isolates. However, the biofilm gene *psl*A was distinguished only on the plasmid of 2 isolates (40%) recovered from minced meat. The *gyr*A gene was perceived in 60% (3/5) of the isolates. Examined isolates improved from minced meat represented the higher MAR index and harbored all tested PMQR besides the biofilm gene *psl*A. Figure 3. Individual agarose gell electrophoresis images for detection of plasmid-mediated quinolone-resistant (PMQR) genes and biofilm-associated gene *psl*A can be seen in Figs. S2. 1- S2. 6.

### Antibacterial activity of EOW and PAA

The antibacterial activity of EOW and the tested concentrations of PAA were determined following the interpretation of the inhibition zone diameters. The results showed that the grouping mixture from EOW and 0.1% PAA provided the higher inhibitory zone for the growing *P. aeruginosa* (17 ± 1.12 mm), followed by the EOW alone (14 ± 1.9 mm), then PAA, which gave an inhibition zone of 12 ± 1.02 mm. These findings determined the higher activity of EOW, particularly when combined with PAA.

### Influence of EOW and PAA on *P. aeruginosa* count

**Fig. 4:**
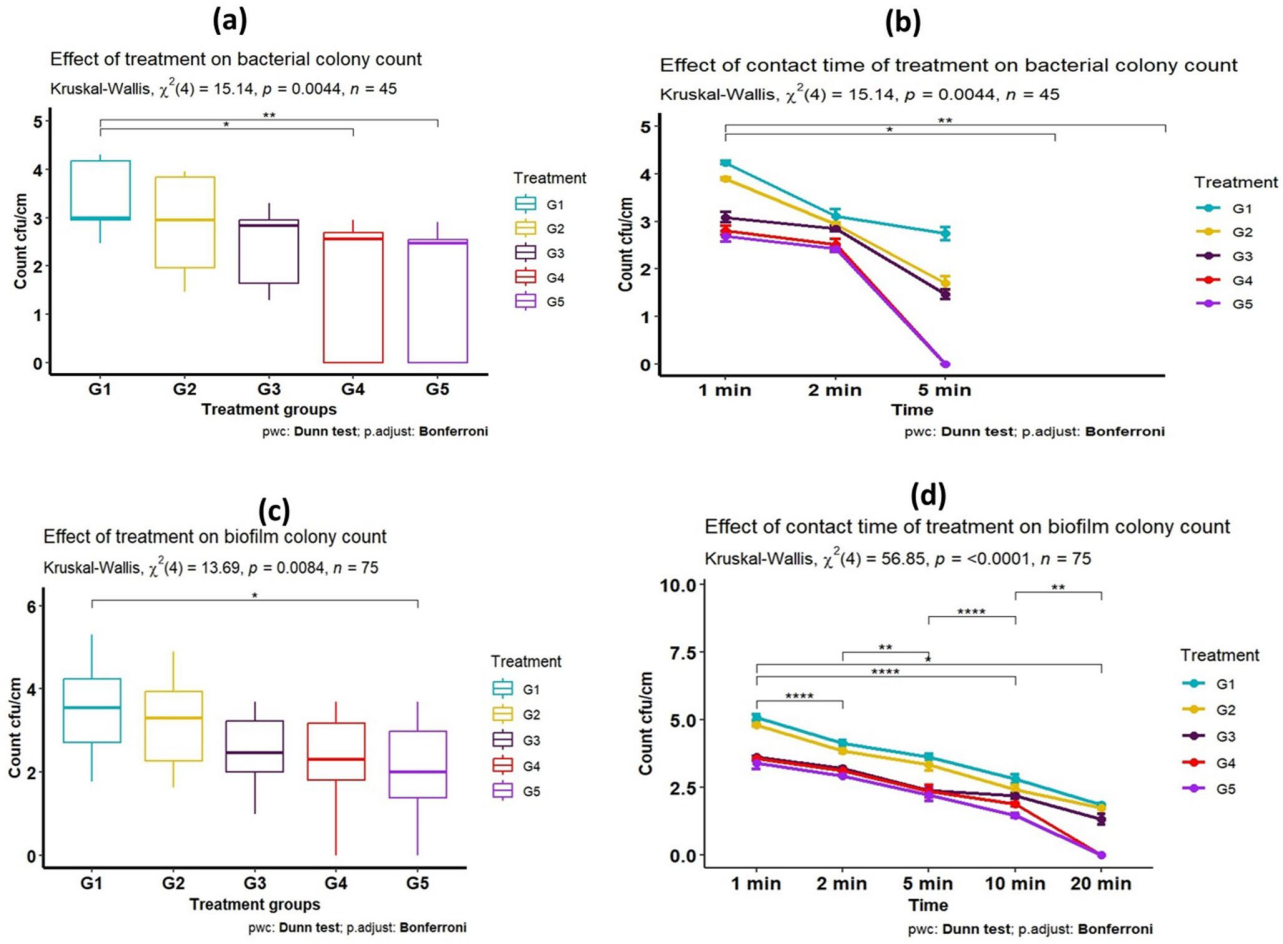
(**a**)- Effect of different treatment on *P. aeruginosa* colony count, (**b**)- Effect of contact time of treatment on *P. aeruginosa* count with initial count (control–ve group) = 7.59 log CFU/cm^2^. (**c**)- Effect of different treatments on *P. aeruginosa* biofilm count and, (**d**)- Effect of contact time of treatment on *P. aeruginosa* biofilm count with initial count (control–ve group) = 9.44 log CFU/cm^2^. G1:treated with 0.1% PAA; G2:treated with 0.3% PAA; G3:treated with EOW; G4:treated with a combined mix of EOW + 0.1% PAA. G5:treated with a combined mix of EOW + 0.3% PAA. * = means a significance difference, ** means moderate significance difference, **** means a highly significance difference

The research experiment revealed that there was a significant discount of the *P. aeruginosa* on SS surfaces with a longer contact time of treatment. Stainless steel surfaces that were contaminated with a suspension containing 8 log10 CFU/mL were treated separately with 0.1% and 0.3% of PAA either alone or in combination with EOW for changed contact times (1, 2, 5, and 10 min). Significant differences (*p* ≤ 0.05) in *P. aeruginosa* count were perceived between the groups treated with the combination mix on one side and other treated groups on the other side. The statistical analysis revealed a significant negative relation between *P. aeruginosa* count and contact-treated times, indicating that longer contact times resulted in a greater reduction in the colony count with a p-value (< 0.001) (Fig. 4).

### Effect of EOW and PAA on *P. aeruginosa* biofilm

The results in Fig. 4 showed that EOW and PAA display a significant activity on *P. aeruginosa* biofilm; there were highly significant differences (*p*-value < 0.05) in *P. aeruginosa* count of G4 and G5 when compared with other treated groups. The *P. aeruginosa* biofilm was completely removed after 20 min in G4 and G5, while in G1, G2, and G3, it was removed after 30 min. The achieved results revealed that the biofilm cell count log CFU/cm^2^ decreased more with improving the contact treatment time (*p*-value < 0.0001).

### Impact of treatments on the relative gene expression levels of quinolone-resistant genes and biofilm-associated gene *psl*A

**Fig. 5:**
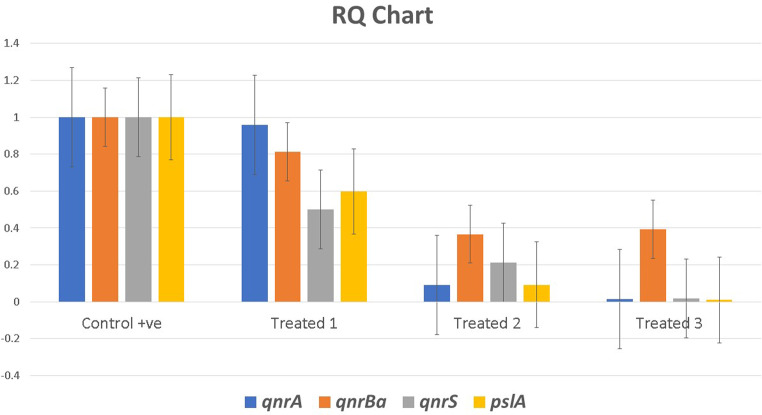
Fold change of the relative gene expression levels of (*psl*A, *qnr*A, *qnr*B, and *qnr*S) concerning the effect of three different treatment groups in comparison to control. Group 1: treated with Per-acetic acid (PAA) 0.1%, Group 2: treated with EOW (pH, 2.1), Group 3: treated with mixed (EOW + PAA 0.1%)

The expression levels of the tested genes *qn*rA, *qnr*B^a^, *qnr*S, and biofilm-associated gene *psl*A were significantly (*p*-value ≤ 0.05) downregulated after 24 h in response to the treatments in all treated groups when paralleled to the control non-treated one. The Kruskal-Wallis rank sum test was conducted to compare the average fold changes in the treated groups, followed by the Dunn test for multiple evaluations. The results revealed that the average fold change of all tested genes significantly declined (*p*-value ≤ 0.05) in treated group 3 (treated with a combination of EOW and PAA 0.1%), then the treated group 2 (EOW alone), and finally treated group 1 (PAA 0.1%). So, these findings suggest a synergistic action of EOW and PAA (Table 3; Fig. 5).

**Table 3:** Fold changes of the relative gene expression levels of the tested genes

Sample No.	Group	16 S rRNA	qnrA		qnrB^a^		qnrS		pslA	
			CT	Fold change	CT	Fold change	CT	Fold change	CT	Fold change
1	Control	14.00	15.79	1	24.73	1	19.91	1	22.81	1
2	Treated 1	13.96	15.81	0.9592	24.99	0.812	20.87	0.50	22.81	0.598
3	Treated 2	17.74	23.00	0.0902	29.92	0.366	25.87	0.214	26.96	0.092
4	Treated 3	14.01	21.83	0.0153	26.09	0.392	25.78	0.0172	28.68	0.010

Treated 1 = group treated with 0.1% per-acetic acid (PAA), Treated 2 = group treated with electrolyzed oxidized water (EOW) water, Treated 3 = group treated with a mix of EOW and PAA 0.1%

## Discussion

*Pseudomonas aeruginosa* is a common spoilage bacteria, principally in nutrient-rich food or on the surface of food processing. It is highly regarded with a higher ability to produce biofilm and higher antimicrobial resistance (Radovanovic et al. 2020). Meat and its products, such as sausage, beef burgers, and luncheons, are rich sources of nutrients and are highly popular in Egypt because they are inexpensive and easy to prepare. In the current work, *P. aeruginosa* was identified in meat products with an overall incidence rate of 24.6% (37 out of 150), which is lower than Farghalyet al. (2022) (37.5%). Only 8% of isolates were identified from burgers, which aligns with Elbayoumi et al. (2021). However, a higher frequency of isolation from burger (26.67%) was recorded by Amal et al. (2014). Previous studies by Farghaly et al. (2022) and Sofy et al. (2017) found an isolation rate of 18% in the total tested sample of luncheons. Minced meat exhibited the highest contamination rate (56%) which is nearly similar to Benie et al. (2017) (53.04%) in West Africa. While lower incidence was reported by Rezaloo et al. (2022) and Elbayoumi et al. (2021) who isolated 7.83% (29/370) and 4% (2/50), respectively, from minced meat. Variations in the isolation rate of *P. aeruginosa* may be accredited to the variation in hygienic procedures during handling preservation and manufacturing of meat products (Abayneh et al. 2019). Furthermore, the high rate of isolation of *P. aeruginosa* in the examined samples may be due to the high capacity of *P. aeruginosa* to adapt to different environmental disorders, ambient temperatures (4 to 42 °C), and moisture (Sofy et al. 2017). Most of *P. aeruginosa* isolates in the current investigation demonstrated biofilm production. The TM assay found that 80% of the total isolates displayed biofilm. Out of them 51% and 29% observed as strong and intermediate biofilms respectevely. However, it is generally less sensitive compared to the TCP method (Elmanamaet al. 2022). The TCP method is considered the most sensitive and reliable for detecting biofilm production. It identified 89.9% of isolates as biofilm producers, with 36.3% producing strong biofilms, 40% intermediate, and 13.6% weak biofilms. This method is often regarded as the gold standard due to its quantitative nature and ability to categorize biofilm production levels more precisely (Abdelraheem et al. 2020). This outcome was consistent with a study conducted by Behzadi et al. (2022), which found that 77.11% of *P. aeruginosa* isolates developed biofilm, with 50% of them being strong producers. Furthermore, an additional study reported that most of the *P. aeruginosa* isolates formed a strong or moderate biofilm (Radó et al. 2017).

Furthermore, 82.3% of the overall *P. aeruginosa* isolates were MDR. In this regard, Islam et al. (2024) reported 100% MDR, while Odoi and Boamah (2021) found MDR in only 43.6% of the isolates. The overall isolates (100%) showed resistance to amoxicillin-clavulanate and cefotaxime, while the isolates completely exposed susceptibility to amikacin. These results are similar to many studies (Araya et al. 2023; Tilahun and Gedefie 2022). The current study demonstrated a minor resistance rate of *P. aeruginosa* to levofloxacin and ciprofloxacin (17% and 23%, respectively), which agreed with Islam et al. (2024), who also recorded a rate of resistance (9.21% and 23.7%) to these antibiotics, respectively. Algammal et al. (2023); Rizk et al. (2024) reported low resistance to quinolone. On the other hand, Behzadi et al. (2022) said that resistance of *P. aeruginosa* against fluoroquinolones was the most common. In the current study, the high resistance of *P. aeruginosa* to β-lactam combinations and cephalosporins may be due to irregular or misuse of these antibiotics, particularly in the veterinary field. Furthermore, the elevated rate of MDR and MAR index of more than 0.2 described in this work indicates the increased risk of contamination and the frequent use of antibiotics (Sandhu et al. 2016). Surprisingly, in the current work, there was no significant connection between biofilm thickness and phenotypic MDR characterization. This result was in agreement with Gajdács et al. (2021); and Zheng et al. (2018) suggests that the antibiotic resistance of biofilm-forming bacterial growth does not depend only on the thickness but may be attributed to additional genetic factors or the presence of persisters (Li et al. 2024). More research studies are needed to understand this relationship well. Biofilm-associated gene *psl*A was detected only in 2/5 (40%) of the tested isolates, which is lower than Kabir et al. (2024) who reported (70.8%), providing an indication that other genes are associated with biofilm formation and support the result of Cho et al. (2018). *P. aeruginosa* infections are commonly treated with quinolones such ciprofloxacin and levofloxacin (Gorgani et al. 2009). However, the misuse as antibiotic therapy frequently led to the development of resistance. *P. aeruginosa* isolates in the current investigation had a lower percentage of entire quinolone resistance (23%) than other antibiotic classes. In gram-negative bacteria, resistance to quinolones is generally produced by chromosomal mutations when the target modification is detected by *gyr*A or *par*C mutations (Hooper 2001). In this study, the PCR-confirmed *P. aeruginosa* isolates were screened for the *gyr*A gene using uniplex PCR assessment. The *gyr*A gene was present on 60% (3 out of 5) of the tested isolates. An earlier study by Abed and Kareem (2021) reported a 44% existence of the *gyr*A gene in the examined *P. aeruginosa* isolates. Furthermore, the appearance of quinolone-resistance genes on plasmids simplifies the horizontal transfer of resistance between bacteria, contributing to the spread of quinolone resistance (Jacoby et al. 2014). Plasmid *qnr* genes were recognized in all examined isolates; however, the gene *qnr*A was the greatest perceived at 80% (4/5) of the examined isolates, followed by *qnr*S (60%), while *qnr*B^a^ was enclosed only in 40% (2/5). A parallel study by Rajaei et al. (2017) in Iran stated that the gene *qnr*A was the main PMQR gene discovered in *P. aeruginosa* isolates. In contrast to Saki et al. (2022), who reported that *qnrB* was the main identified gene. An additional study by Molapour et al. (2020) reported the absence of these genes in all isolates. In the current study, the examined isolates recovered from minced meat were characterized by the higher MAR index and harbored all stated PMQR and biofilm genes, suggesting that *P. aeruginosa* strains improved from minced meat represent a higher-risky source for contamination than luncheon and burgers. The study assessed the antibacterial activity of EOW and PAA against *P. aeruginosa*. PAA alone produced a smaller inhibition zone of 12 ± 1.02 compared to EOW alone, which showed significant antibacterial activity with an inhibition zone of 14 ± 1.9 mm. The combination of EOW with 0.1% PAA produced the largest inhibition zone (17 ± 1.12 mm), indicating a synergistic interaction. EOW can display potent antimicrobial properties through its high oxidation-reduction potentials (ORP); it can denature proteins and damage microbial DNA (Rahman et al. 2016). PAA is a potent oxidizing agent that can oxidize the cell components, causing cell damage; also it may interfere with the key metabolic pathways, inhibiting bacterial growth and replication (Block 2001). So, when combined, EOW and PAA can ensure a synergistic effect, improving their overall antimicrobial activity. Many strategies were developed to prevent *P. aeruginosa* biofilm formation for industrial purposes. The use of chemical sanitizers can be harmful to human health and can also lead to resistance by microorganisms (Liu et al. 2021). Therefore, in order to replace chemical antimicrobial agents, scientists must discover safe, natural, efficient, and economically substitutes. The study investigated the effect of EOW and PAA on *P. aeruginosa* bacterial and biofilm cell count using Stainless steel (SS) surfaces, as they are generally used in food processing and industry. The initial count of P. aeruginosa was 7.59 log CFU/cm². The immersion of SS surfaces in treatment solutions of PAA 0.1% (G1), PAA 0.3% (G2), EOW (pH 2.1) (G3), a mixture of EOW and peroxyacetic acid 0.1% (G4), and a mixture of EOW and peroxyacetic acid 0.3% (G5) for 1, 2, 5, and 10 min resulted in a reduction of *P. aeruginosa* count to 4.23 ± 0.06, 3.89 ± 0.05, 3.08 ± 0.18, 2.81 ± 0.15, and 2.68 ± 0.18 log CFU/cm^2^, respectively, after 1 min. while after 2 min of contact, there were clear significance differences (*P* ≤ 0.05) in *P. aeruginosa* count between all groups. However, after 5 min, G4 and G5 showed complete inhibition of *P. aeruginosa* count, while a clear count reduction was seen in G1, G2, and G3. The *P. aeruginosa* count was completely inhibited (not detected) in all groups after 10 min. Our result agreed with Liu et al. (2023),, who found that EOW reduced *P. aeruginosa* from 7.87 to 2.51 log CFU/mL in 8 min. There was a relation between contact time and *P. aeruginosa* reduced count, a *p*-value (< 0.001), where more count reduction was parallel with increasing exposure time. However, biofilm needs more time to be eradicated. As shown in Fig. 4, groups G4 and G5 showed complete inhibition of *P. aeruginosa* biofilm after 20 min, while biofilm was reduced in G1, G2, and G3. The *P. aeruginosa* biofilm was completely inhibited (not detected) in all groups after 30 min. Martín-Espada et al. (2014) reported that PAA is effective against *P. aeruginosa* arranged biofilms on the polystyrene surfaces when used with a concentration of 1.61% and a contact time of 15 min, resulting in 100% inhibition of the bacterial population. The variation of the result may be affected by the type of contact surface and temperature (Abdallah et al. 2015). The bactericidal effect of EW is focused on losing bacterial membrane integrity for different foodborne pathogens (Medina-Gudiño et al. 2020). Thus, electrolyzed water is considered a significant method for destroying and eliminating bacterial biofilms that are important for food safety and public health (Yan and Daliri 2021). The combination of PAA and EOW revealed a significant discount in *P. aeruginosa* arranged biofilm (*p*-value < 0.0001). Specifically, with higher concentrations of PAA and EOW after 20 min in treated groups (G4 and G5), compared to 30 min in other groups (G1, G2, and G3). This suggested a synergistic action of PAA and EOW enhancing the antimicrobial efficacy against *P. aeruginosa* arranged biofilms (Iram et al. 2021; Yan and Daliri 2021; Liu et al. 2023).

The expression levels of resistance genes in bacterial isolates may vary depending on the host and/or the surrounding environmental conditions (Sandra Georgina et al. 2020). Also, the stability of the plasmid may be affected by environmental conditions such as acidic hydrogen ion concentration (pH), which correspondingly can affect the expression level of resistance genes in bacteria (Jacoby et al. 2014). In the current work, EOW showed a significant decrease in the fold change of relative expression levels of quinolone resistance genes (*qnr*) and the biofilm-related gene *psl*A, especially when combined with PAA. This outcome appears to be in agreement with Shimamura et al. (2024), who stated that EOW can inhibit the expression of virulence factors. Concerning quinolone-resistant genes, which were significantly decreased after treatment with EOW in this study. Rodríguez-Martínez et al. (2011) reported that acidic pH can result in changes in the expression level of quinolone-resistance genes in bacteria. Also, Correia et al. (2017) stated that acidic environments can potentially improve the action of efflux pumps, reducing the intracellular concentration of quinolones and thus increasing resistance. Moreover, it was reported that PAA is a disinfectant that can have an impact on the bacterial cells and possibly may cause an effect on the expression level of antibiotic-resistance genes. In the present study, treatment with PAA 0.1% resulted in a smaller decrease in the fold change (0.959, 0.812, 0.50, and 0.598) of *qnr* gene expression levels compared to treatment with EOW, which showed fold changes of (0.0902, 0.366, 0.214, and 0.092). A previous study by Biswal Basanta et al. (2014) reported that the prevalence of antibiotic resistance genes (ARGs) carried by resistant strains of *E. coli* decreased by an average of 47% with a fold change 0.430 when exposed to PAA. However, another study conducted by Yin et al. (2023) stated that PAA disinfection may pose a risk of spreading antimicrobial resistance (AMR) in the natural environment. However, that study revealed that even after PAA disinfection, the antimicrobial-resistant *E. coli* still carried a significant amount of antimicrobial resistance genes (ARGs), potentially leading to the transmission of AMR in the natural environment. Therefore, these findings suggested the need to explore the combined action of electrolyzed-oxidizing water (EOW) with PAA, which is supported by Kondo et al. (2006).

## Conclusion

The existing study demonstrated that EOW, especially when used in combination with PAA, exhibits strong antibacterial activity against *P. aeruginosa* and significantly decreases the expression level of quinolone-resistant genes, which suggests that such combinations could be valuable in developing more effective antibacterial treatments and strategies to combat resistant bacterial infections and to control the spread of plasmid-mediated quinolone resistance. The findings also suggest that PAA and EOW are effective agents for biofilm control on SS surfaces. The significant reduction in *P. aeruginosa* counts with increased contact time and higher concentrations of PAA highlights their potential application in various industrial and clinical settings. The complete removal of biofilm in groups treated with a combined mix of PAA and EOW during only 20 min demonstrates the efficiency of these treatments in achieving rapid biofilm eradication.

## Electronic supplementary material

Below is the link to the electronic supplementary material.

Supplementary Material 1

Supplementary Material 2

## Data Availability

No datasets were generated or analysed during the current study.
