# Supplementary material for: Synergistic effect of electrolyzed oxidized water (EO) and peroxyacetic acid on plasmid-mediated quinolone resistance genes of Pseudomonas aeruginosa
Source: World J Microbiol Biotechnol. 2025 Jun 14;41(6):199. doi: 10.1007/s11274-025-04384-w (PMC12165879; doi:10.1007/s11274-025-04384-w)
Supplement: Supplementary file 1 — Supplementary Material 1 [file 11274_2025_4384_MOESM1_ESM.docx]

**Individual agarose gell electrophoresis images for detection of plasmid-mediated quinolone-resistant (PMQR) genes and biofilm-associated gene *psl*A**


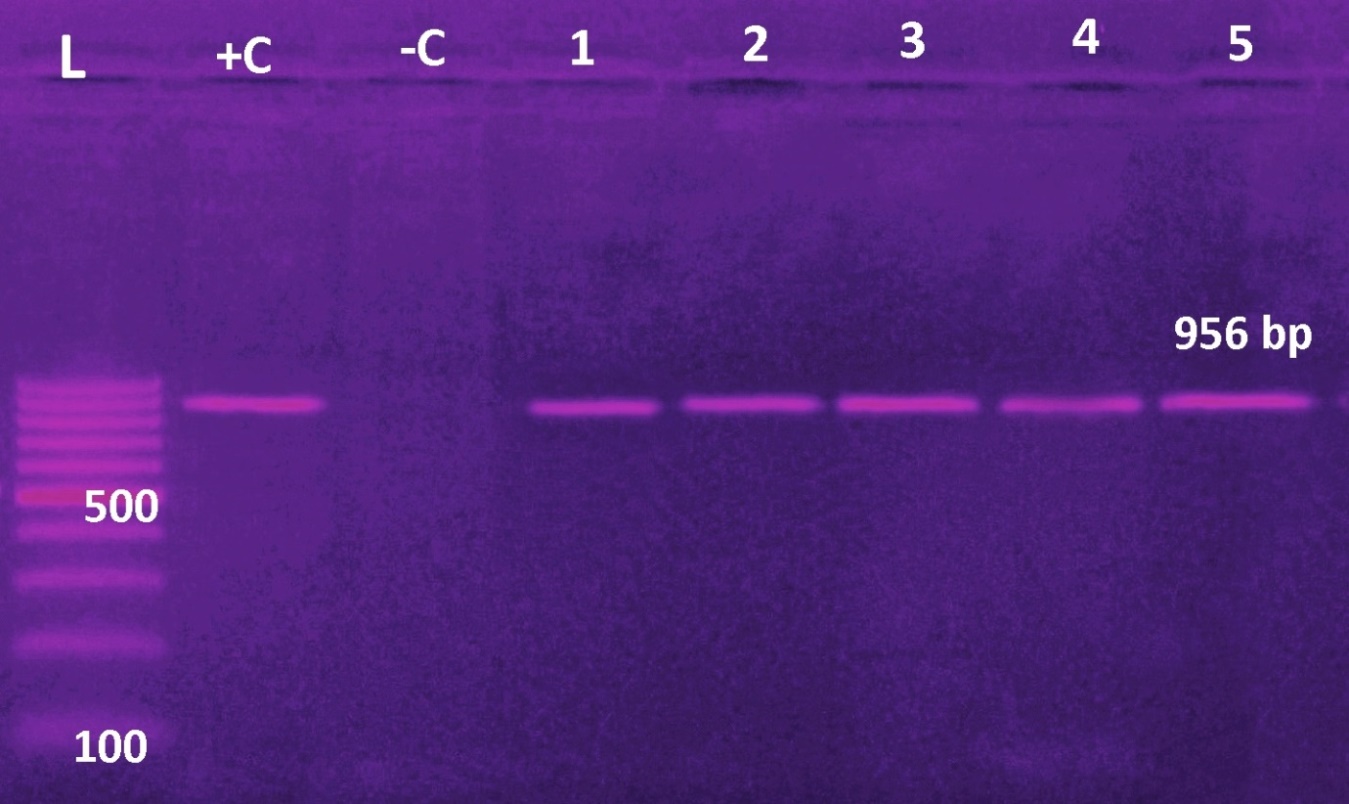


**Fig. S2. 1. A garose gell electrophoresis for 16S*r*RNA**


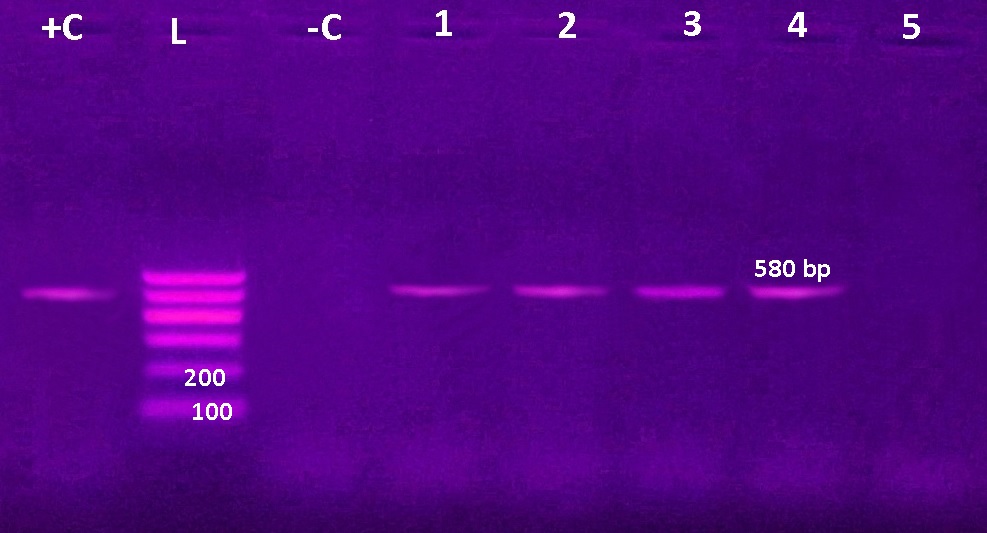


**Fig. S2. 2. A garose gell electrophoresis for *qnr*A**


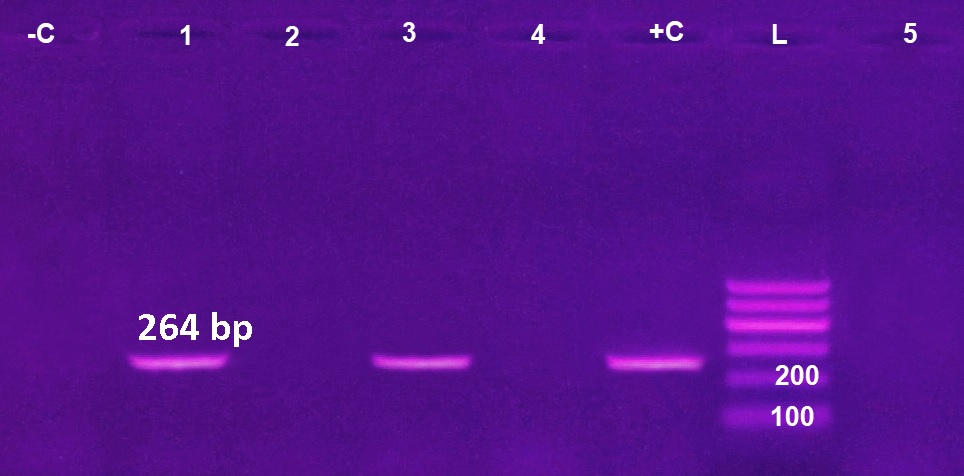
 **Fig. S2. 3. A garose gell electrophoresis for *qnrB***


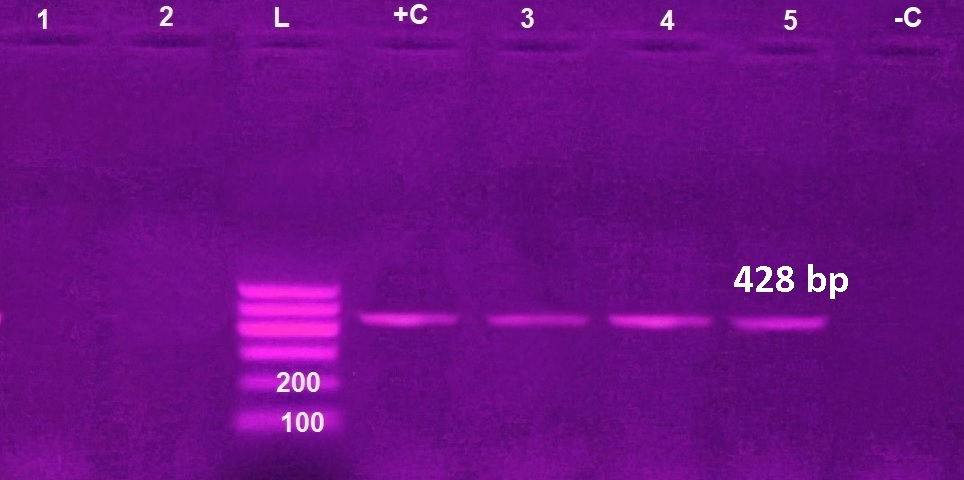


**Fig. S2. 4. A garose gell electrophoresis for *qnrS* gene**


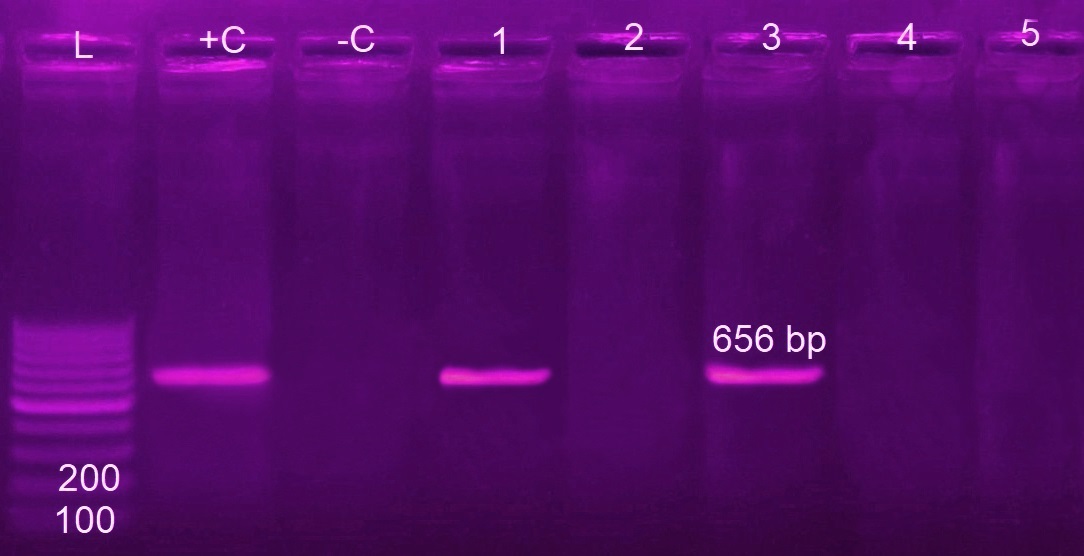


**Fig. S2. 5. A garose gell electrophoresis for *Psl*A gene**

**
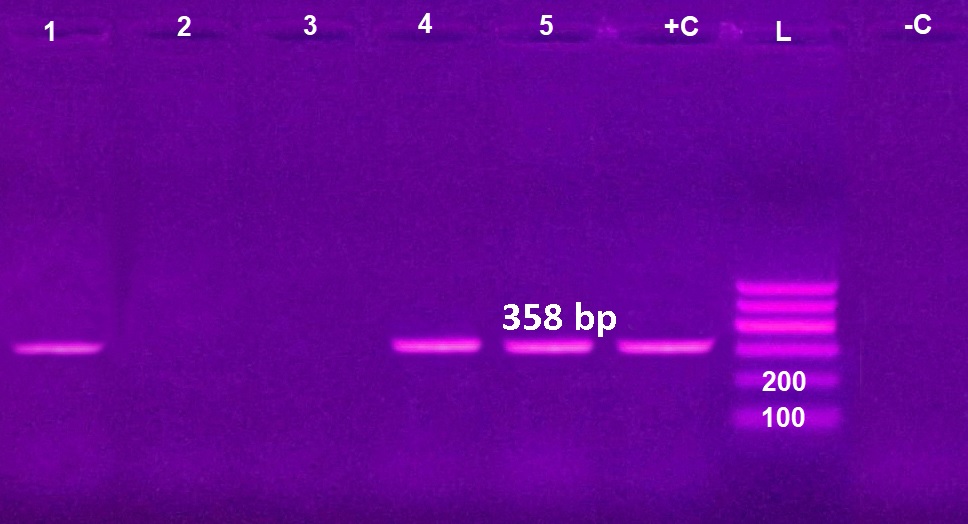
**

**Fig. S2. 6. A garose gell electrophoresis for *gyr*A gene**
