# Supplementary material for: Synergistic effect of electrolyzed oxidized water (EO) and peroxyacetic acid on plasmid-mediated quinolone resistance genes of Pseudomonas aeruginosa
Source: World J Microbiol Biotechnol. 2025 Jun 14;41(6):199. doi: 10.1007/s11274-025-04384-w (PMC12165879; doi:10.1007/s11274-025-04384-w)
Supplement: Supplementary file 2 — Supplementary Material 2 [file 11274_2025_4384_MOESM2_ESM.docx]

**Individual images for phenotypic detection of biofilm-forming ability**


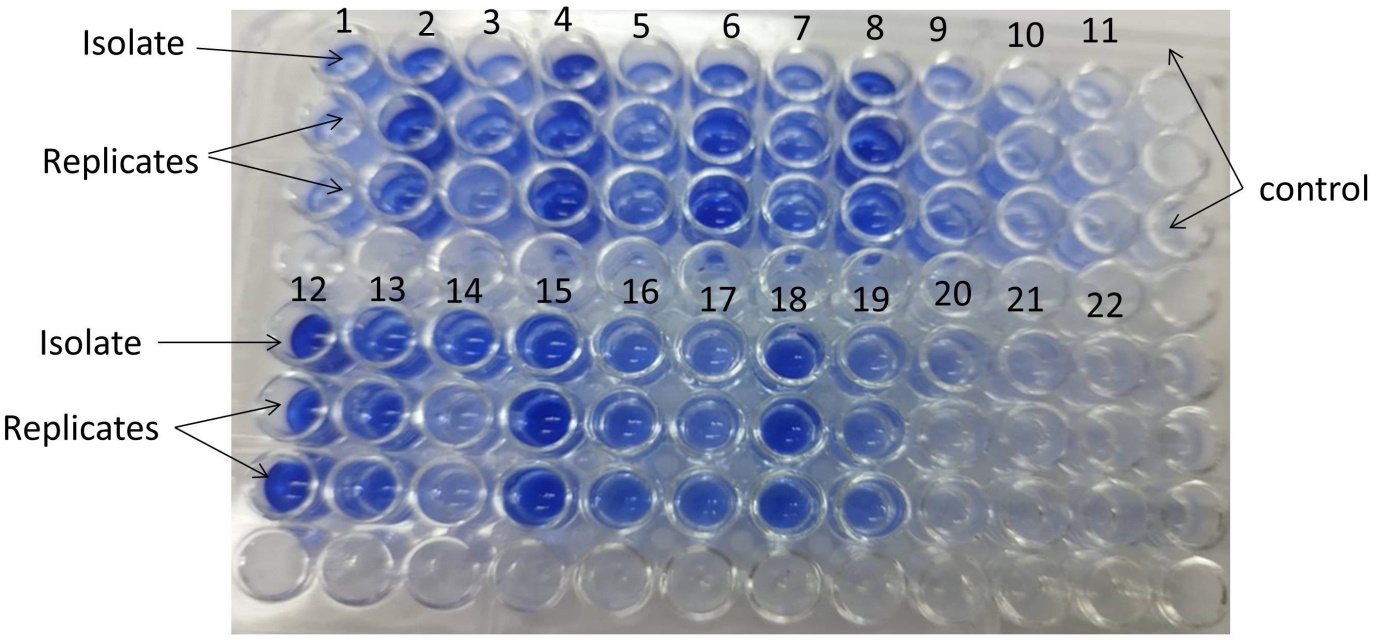


Fig. S1. 1. Micro-titre plate after staining the biofilm with crystal violet stain showing the biofilm formation for the tested isolates and their replicates.


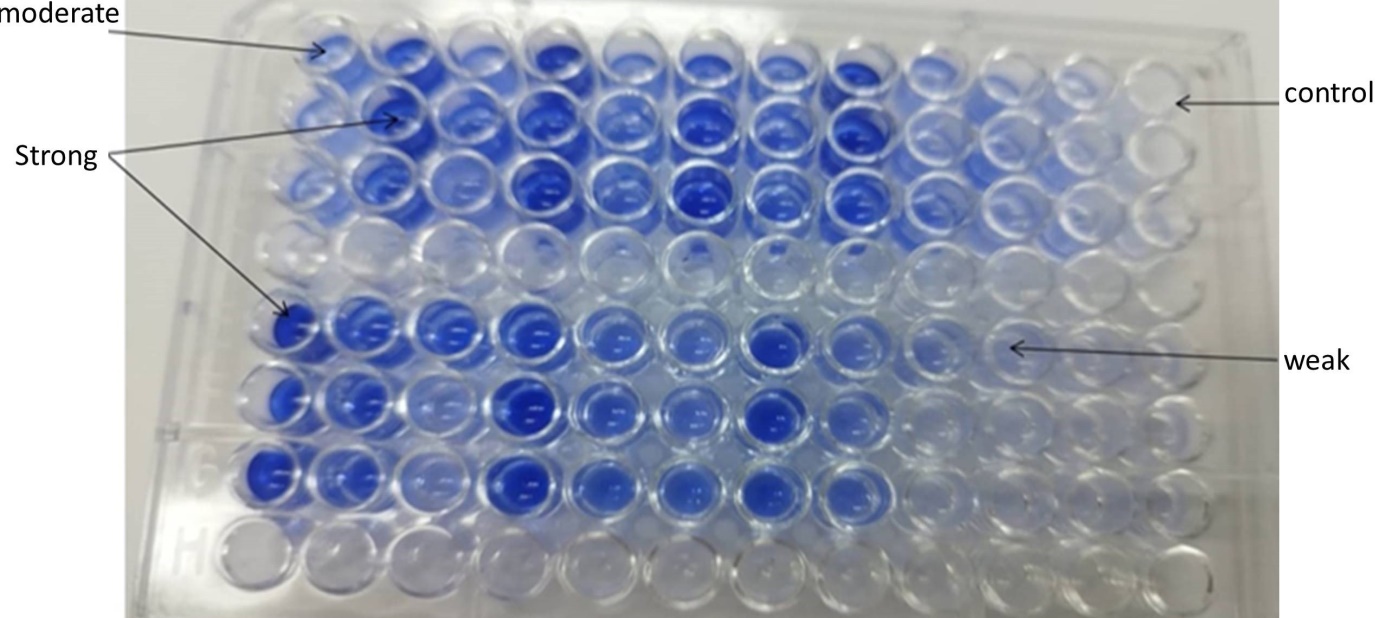


**Fig. S1. 2. Micro-titre plate after staining the biofilm with crystal violet stain showing the biofilm density (strong, moderate, and weak) biofilm formation for the tested isolates and control.**


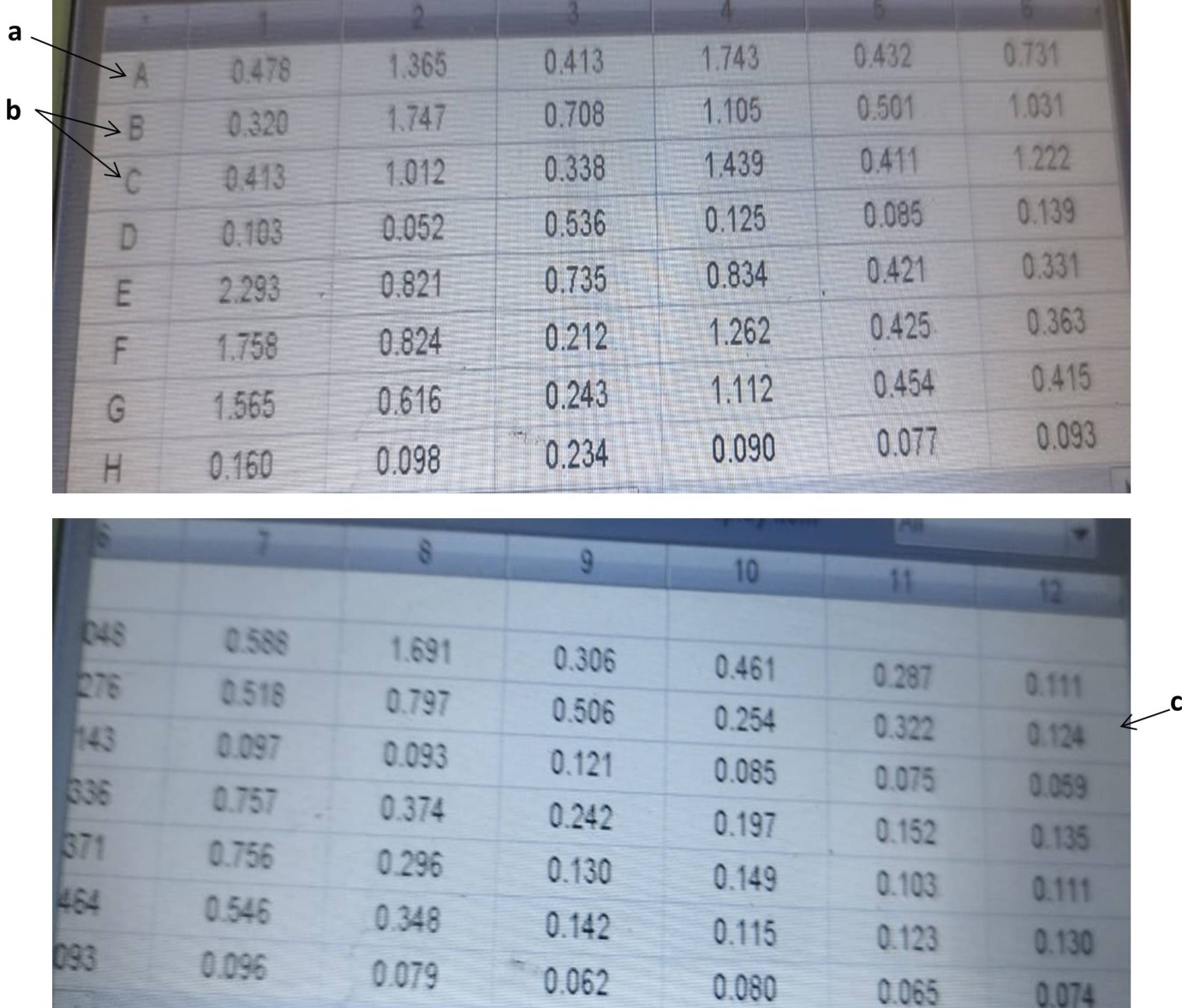


Fig. S1. 3. Reading of the optical density of the tested isolates on ELSA reader

a: optical density for isolates; b: optical density for replicate; c: optical density for control

Table S1. Biofilm construction classification for optical density reading results

| **Cut-Off Value** | **Mean of OD values** | **Biofilm density** |
| --- | --- | --- |
| ODc | 0.14 | None biofilm producer |
| ODc < OD ≤ 2 ODc | 0.14< OD ≤ 0.28 | Weak biofilm producer |
| 2 ODc < OD ≤ 4 ODc | 0.28 < OD ≤ 0.56 | Intermediate biofilm producer |
| 4 ODc < OD | OD ≥ 0.56 | Strong biofilm producer |

Optical density cut-off value (ODc); ODc = average OD of the negative controls + (3 × SD of negative control)


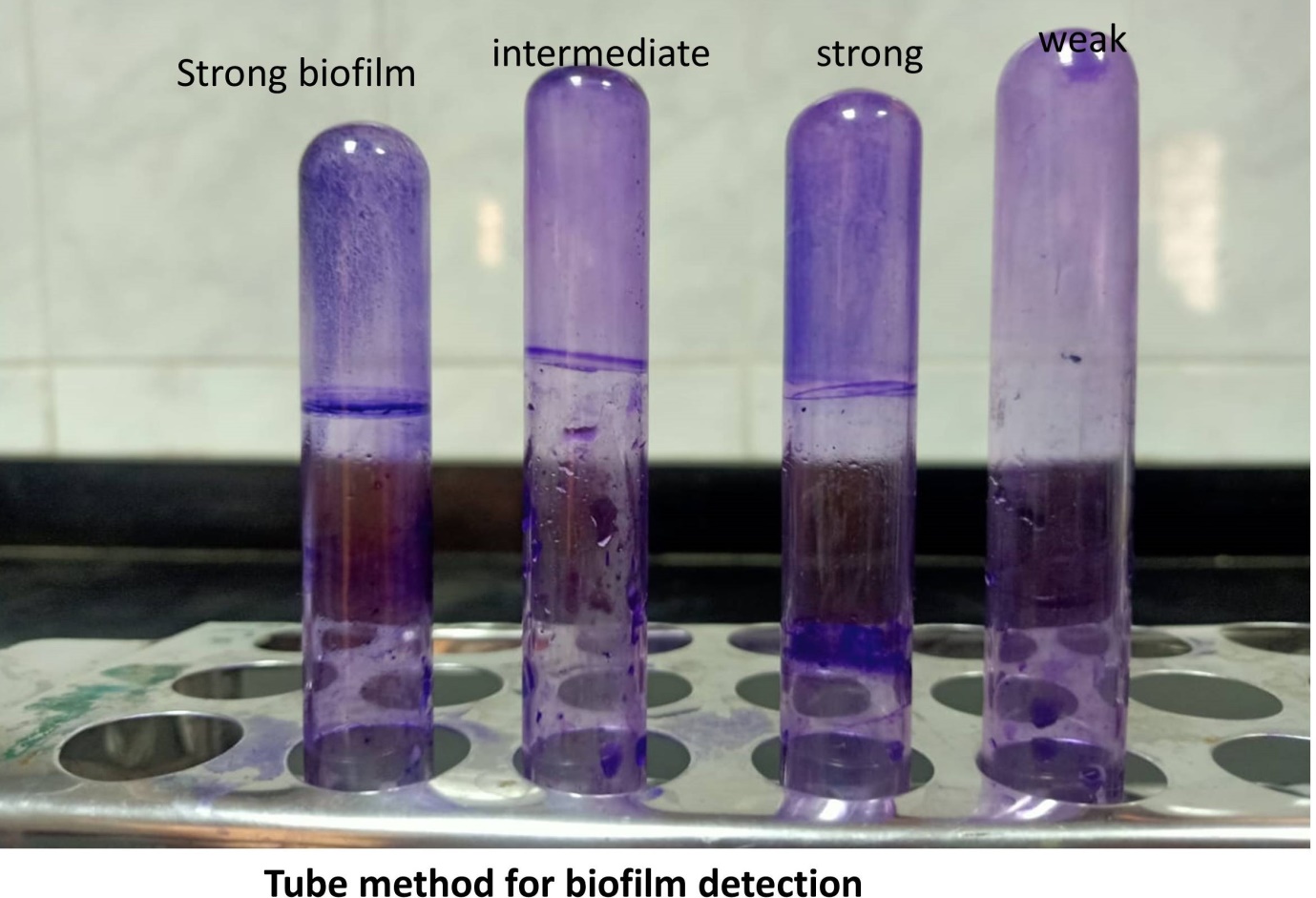


**Fig. S1. 4. Tube method for biofilm detection showed biofilm formation concentration ( strong, intermediate, and weak ).**
